# The impact of the COVID-19 pandemic on accessing HIV care: A case report

**DOI:** 10.4102/safp.v63i1.5344

**Published:** 2021-09-27

**Authors:** Ramprakash Kaswa

**Affiliations:** 1 Department of Family Medicine and Rural Health, Faculty of Health Sciences, Walter Sisulu University, Mthatha, South Africa

**Keywords:** COVID-19, pandemic, HIV, ART, lockdown, morbidity and mortality

## Abstract

The coronavirus disease 2019 (COVID-19) pandemic has had an enormous impact on the provision of human immunodeficiency virus (HIV) services amongst people living with HIV. Many people have adopted different health-seeking behaviour in alignment with the lockdown provisions during the COVID-19 pandemic. These lockdown regulations have had a huge impact on healthcare access for people on chronic medication. The disruption of antiretroviral therapy (ART) has a profound effect on HIV-associated morbidity and mortality. The impact on HIV programmes as a result of the interruption in ART could be bigger than the HIV pandemic alone.

## Introduction

The impact of the coronavirus disease 2019 (COVID-19) pandemic on the world is beyond our imagination. The perfect storm of economic, health and social crisis was created by the COVID-19 pandemic.^1^ As most of the countries around the globe underwent lockdown periods, access to healthcare was severely impacted because of an interruption of supply, a diversion of resources and the overwhelm of healthcare systems.^1,2^

Like other countries around the world, South Africa also implemented a lockdown to slow the transmission rate and prepare the healthcare system for the COVID-19 pandemic. These provisions have had enormous consequences on healthcare service access for patients with chronic diseases, particularly for those living with HIV and AIDS.^3,4^ South Africa accounts for about one-fifth of the global HIV burden and has the world’s largest antiretroviral therapy (ART) programme.^5^ In recent years, South Africa has made enormous progress towards HIV management.

In the context of the COVID-19 pandemic, many patients changed their health-seeking behaviour, even to acquire essential drugs such as ART.^1,5^ Poor adherence to ART is associated with increased morbidity and mortality amongst people living with HIV.^6^ The purpose of this case report is to understand the profound effect of the COVID-19 pandemic on the continuity of care amongst people living with HIV.

## Case

A 51-year-old female patient was brought to the Accident and Emergency (A&E) department of our regional hospital by her relatives with complaints of cough, fever and an inability to eat and walk. She was screened for severe acute respiratory syndrome (SARS) COVID-19 becausee she presented with possible COVID-19 symptoms. She tested positive for the rapid antigen test for SARS COVID-19 and the nasopharyngeal swab for severe acute respiratory syndrome coronavirus 2 (SARS-CoV-2). A reverse transcription-polymerase chain reaction (RT-PCR) test was sent for laboratory testing.

**FIGURE 1:**
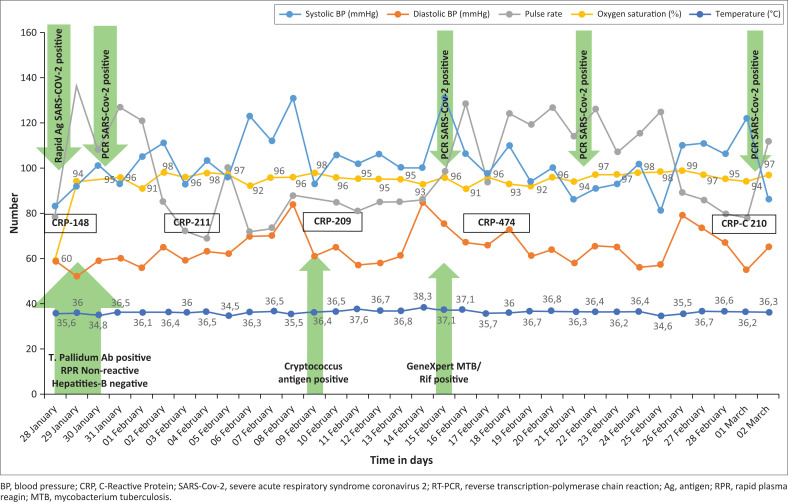
Vital sign and laboratory results between 28th January 2021 and 3rd March 2021.

On presentation to the A&E department, she was chronically ill-looking, dehydrated, with blood pressure (BP) of 83/59 mm of mercury (mmHg), a pulse of 78/min, oxygen saturation of 60% at room air and a body temperature of 37.3 °C. Figure 1 demonstrates her vital signs and laboratory results between 28th January 2021 and 3rd March 2021. An examination of the respiratory system revealed coarse crackles with decreased air entry in both lung fields. Cardiovascular, gastrointestinal, genitourinary and nervous system examinations were within normal limits. Upon oral examination, an active lesion of oropharyngeal candidiasis was noticed.

According to the patient, she lived alone and had some support from distant relatives, who occasionally visited her. She was on ART since 2014 after an HIV diagnosis and, until the start of the COVID-19 pandemic lockdown in March 2020, she was well compliant with her HIV treatment. During the lockdown, the local clinic was closed for a few days during April 2020 because of one of the staff members contracting the COVID-19 infection. As a result of the fear of COVID-19, she avoided visits to the health facility and defaulted on her ART from April 2020. The viral load of her laboratory results is summarised in Table 1.

**TABLE 1:** Laboratory results of viral load between 2015 and 2021.

S. No	Date and year	Viral load	Absolute CD4
1	8th April 2015	Lower than detectable limit	143 cells/mm^3^
2	4th August 2017	Lower than detectable limit	No record
3	23rd January 2018	Lower than detectable limit	No record
4	9th January 2019	Lower than detectable limit	No record
5	30th January 2020	Lower than detectable limit	No record
6	28th January 2021	1 939 662 Copies/mL	7 cells/mm^3^

A clinical diagnosis of COVID-19 pneumonia and HIV with defaulted ART was made. She was rehydrated with normal saline and started with dexamethasone 6 mg, low molecular weight heparin 40 mg, nystatin, a stat dose of ceftriaxone 1 mg and baseline blood was sent for a laboratory test. She was admitted to the COVID-19 ward. Oxygen saturation was improved from 60% on room air to 94% on a face mask with oxygen. The following day, her blood investigation showed haemoglobin at 12.9 mmol/dL, a white cell count of 3.54 L × 109/L, platelets at 64 L × 109/L, SARS-CoV-2 RT-PCR positive, C-Reactive Protein (CRP) at 148 mg/L, cluster of differentiation 4 (CD4) count of 7 cells/mm^3^, viral load of 1 939 662 Copies/mL, Hepatitis-B antigen-negative, *Treponema pallidum* antibody positive and rapid plasma reagin (RPR) nonreactive. A chest X-ray showed bilateral consolidations and she was unable to produce sputum. After a negative tuberculosis (TB) screening, she re-initiated ART and started tuberculosis prevention therapy (TPT) and co-trimoxazole prophylaxis. She improved clinically, initially, but presented with confusion after a week. Serum cryptococci antigen (CrAg) tested positive but the laboratory results of cerebral spinal fluid (CSF) reported negative for meningitis. The fluconazole protocol for cryptococci was started but she continued to deteriorate. The following week, she tested positive for GeneXpert mycobacterium tuberculosis (MTB)/rifampicin sensitivity and TPT was stopped whilst anti-TB treatment was initiated. The clinical diagnosis was revised to COVID-19 pneumonia, TB immune reconstruction inflammatory syndrome (IRIS) and HIV. As demonstrated in Figure 1, her PCR test for SARS-Cov-2 remained positive on the 15th, 23rd and 32nd days since she was first tested and the CRP constantly increased over time. She continuously deteriorated despite optimum treatment and expired on 3rd March 2021.

## Discussion

The COVID-19 pandemic has had a substantive negative impact on people living with HIV.^1^ They are less likely to seek healthcare because of fear and uncertainty surrounding the COVID-19 pandemic, especially during lockdown when restrictions on movement are enforced.^5^ The unannounced closure of clinics because of COVID-19 infections amongst healthcare staff reduced service availability. Other measures, such as social distancing, that were put in place to combat the spread of COVID-19, disrupted the progress made by many existing healthcare programmes, including the HIV continuum of care.^5,7^ This disruption in healthcare access caused by COVID-19 has adverse health consequences for people beyond the health concerns caused by COVID-19 itself.^5^

A survey conducted by the Human Science Research Council (HSRC) amongst people living with HIV reported that about 13% of people did not have access to their medication during the COVID-19 lockdown.^8^ Interruptions of ART amongst people living with HIV can precipitate virological failure and make them vulnerable to opportunistic infections.^6,9^ Our case reports the virological failure and severe immunosuppression with opportunistic infections. She was well compliant during the last 6 years with an undetectable viral load, but after the disruption of ART for the last 8 months, she presented with a viral load of 1 939 662 Copies/mL and a CD4 count of 7 cells/mm^3^. These disruptions in HIV programmes because of the COVID-19 pandemic have had a substantial impact on HIV-associated morbidity and mortality.^10^ These negative impacts of the COVID-19 pandemic on access to healthcare services have started to emerge.^5^

According to national department of health guideline positive antigen test amongst symptomatic patient in high community prevalence is confirmed as COVID-19 and follow up RT-PCR test is not recommended.^11^ Our patient’s SARS-CoV-2 RT-PCR test on day 32 remained positive after the initial detection. Several studies reported prolonged and recurrent detection of viral ribonucleic acid (RNA) by the RT-PCR tests from symptomatic and asymptomatic COVID-19 patients.^9,12,13^ Although most study reported that persistent viral RNA shading did not increase the risk of transmission but further research needed amongst people living with HIV. Immunosuppression also plays a major role in recurrent detection and prolonged viral shedding. There are several case reports demonstrating the isolation of infectious viral particles from immunosuppressed patients after several weeks to months from the initial infection.^9,12^ A non-Hodgkin’s lymphoma patient reported a positive nasopharyngeal swab SARS-CoV-2 RT-PCR after 8 months from the initial infection.^13^ A study conducted by Western Cape Department of Health in collaboration with the National Institute for Communicable Diseases not only reported HIVas an independent risk factor for mortality irrespective of viral load and CD4 count but study also accepted that it could be overestimated because of residual confounding factors.^14^ One can speculate that an immunosuppressed patient is likely to have different pathogenesis of the COVID-19 than in immunocompetent individuals.

According to a survey conducted on HIV care in 65 South African primary care facilities, the fear of contracting COVID-19 was the main reason for not visiting healthcare facilities during the lockdown period.^5^ The current case subject also interrupted her ART because of the fear of contracting COVID-19 at the healthcare facility and ended up defaulting on her HIV treatment. Healthcare workers need to establish a clear engagement protocol to overcome the challenges involved in attending healthcare facilities during the lockdown period. Other reasons for not visiting healthcare facilities that were highlighted in the survey were disruption to public transportation services and stay-at-home orders during lockdown.^7^ Some patients sought delayed care, whilst others no longer visited the healthcare facility because of a misinterpretation of the recommended changes that only essential visits be permitted during the COVID-19 lockdown.^10^ During the COVID-19 pandemic healthcare facilities adopted new approaches of healthcare management including the implementation of minimum 2 month ART dispensation or refer the patient to external pickup point. When people have restricted access to healthcare facilities because of COVID-19 lockdown regulation than alternative approach is bringing the healthcare services to the doorstep of people. Home delivery of chronic medication including ART by community health workers in Western Cape province is such an example of strengthening chronic medication adherence during COVID-19 lockdown.^15^

## Conclusion

The study’s findings suggest that the poor access to healthcare services during the COVID-19 lockdown has had a serious impact on HIV care. In addition, immunodeficiency because of poor ART adherence amongst people living with HIV increases their vulnerability for severe COVID-19 and is associated with prolonged viral shedding. It is crucial to monitor patients with ART to ensure their adherence to the treatment and all efforts should be made to encourage people to seek healthcare to address the current and potential future COVID-19 outbreak.
